# Unconvincing statistical and functional inferences: reply to Catmur

**DOI:** 10.3389/fnhum.2014.00887

**Published:** 2014-10-31

**Authors:** John Michael, Kristian Sandberg, Joshua Skewes, Thomas Wolf, Jakob Blicher, Morten Overgaard, Chris Frith

**Affiliations:** ^1^Department of Cognitive Science, Central European UniversityBudapest, Hungary; ^2^Interacting Minds Centre, Aarhus UniversityAarhus, Denmark; ^3^Cognitive Neuroscience Research Unit, Hammel Neurorehabilitation and Research Centre, Aarhus UniversityAarhus, Denmark; ^4^Institute of Cognitive Neuroscience, University College LondonLondon, UK; ^5^Cognitive Neuroscience Research Unit, Center of Functionally Integrative Neuroscience/MindLab, Aarhus UniversityAarhus, Denmark; ^6^Department of Communication and Psychology, Centre for Cognitive Neuroscience, Aalborg UniversityAalborg, Denmark; ^7^Wellcome Trust Center for NeuroimagingLondon, UK

**Keywords:** mirror neuron, transcranial magnetic stimulation, action understanding, perception, premotor cortex, action perception

In a recent commentary published in *Frontiers in Human Neuroscience*, Catmur (2014) raises several important questions for discussion about a study we published earlier this year (Michael et al., 2014). In the following, however, we point out that her criticism is based upon two inferences that we do not find convincing, and we maintain that the conservative interpretation offered in our original article is more appropriate than Catmur's alternative interpretation.

In our study, we used offline continuous theta-burst stimulation (cTBS) to investigate whether regions of premotor cortex (PMC) play a causal role in action understanding. Participants received cTBS over the hand and lip areas of left PMC, in separate sessions, before completing a pantomime-recognition task in which half of the trials contained pantomimed hand actions, and half contained pantomimed mouth actions. The results revealed a double dissociation: Participants were less accurate in recognizing pantomimed hand actions after receiving cTBS over the hand area than over the lip area and less accurate in recognizing pantomimed mouth actions after receiving cTBS over the lip area than over the hand area. We argued that this finding constrains theories of action understanding by showing that somatotopically organized regions of PMC contribute causally to action understanding.

However, as Catmur observes, a further aim of the study was to shed light upon the specific functional role of the targeted premotor neural populations, i.e., whether they contribute to action understanding by encoding kinematic information about observed actions, or by encoding the proximal or distal goals (e.g., to grasp a cup or to drink) of those actions. We therefore devised three separate tasks to probe different components of action understanding. The simplest of the three tasks required participants to identify still frames from brief videos of pantomimed actions. This task thus probed a perceptual component of action understanding, i.e., the ability to process kinematic features of observed actions. An intermediate task required them to select which of three objects complemented a brief video of a pantomimed action, thus probing their ability to identify the proximal goal of an observed action. The most complex task required them to select which of three objects complemented a brief video of a pantomimed action in a context-sensitive manner, thus probing their ability to identify the distal goal of an observed action. The results showed no significant difference among these three tasks. Catmur concludes that the most adequate interpretation of the data would be one that identifies the functional contribution of the targeted neural populations as whatever is common to the three tasks—and this is the encoding of kinematic information. As she puts it:

“Recall that all three tasks required action perception: since performance on all three tasks was impaired, these data suggest that mirror neuron areas are involved, *not* in higher-level processes such as matching an action to its goal object or selecting the relevant object for that action in a given context, but instead in a lower-level process of action perception” (Catmur, 2014, p. 2).

Note, however, that this constitutes an inference from the absence of evidence to evidence of absence. But the absence of a significant interaction does not warrant the inference that the impact of magnetic stimulation for all tasks is the same; it simply means that we did not find evidence that it is different. This may have been due to various reasons, one important candidate being lack of statistical power. A further possibility is that any neurons contributing differently among the three levels of complexity are not somatotopically distributed, and are affected more or less equally at the two stimulation sites. In view of our ignorance on this matter, we believe that the more conservative conclusion we originally offered (acknowledging the possibility that the targeted areas specialize in processing low-level kinematic information) is the most appropriate one:

“Given the absence of any three-way interaction of TMS site, video type, and complexity level (simple, inter-mediate, complex), our results do not permit any inferences about the hierarchical level at which premotor regions contribute to action understanding (i.e., whether these areas specifically encode low-level kinematics, proximal goals, or distal goals). One possibility is that the areas we targeted encode low-level kinematic information about observed movements and that this kinematic information is relevant for tasks of varying complexity” (Michael et al., 2014, p. 970).

It is also worth noting that Catmur draws a second inference as well: from the claim that mirror neuron areas may be involved in “action perception” to the further claim that they are not involved in “higher-level processes such as matching an action to its goal object or selecting the relevant object for that action in a given context.” This inference depends upon the radically modularist premise that it is not possible for some of the processes underpinning action perception sometimes to contribute to the process of making judgments about the (proximal and distal) goals of observed actions. We see no reason to accept this premise. As a result, while we agree with Catmur's assessment that the areas in question possibly (even likely) specialize in a relatively low-level process (encoding kinematic information about observed actions), we also suggest that this low-level process can contribute to the process of making judgments about the (proximal and distal) goals of observed actions.

Clearly there is a risk of falling into mere terminological quibbling about the terms “action perception” and “action understanding,” and Catmur is right to observe that such quibbling has hampered research in this area. It was in part in order to avoid this that we invoked the more precise tripartite distinction among kinematics, proximal goals and distal goals [borrowed from Hamilton and Grafton (2007)]. Although our results did not ultimately resolve the question as to the hierarchical level at which the areas targeted in our study contribute to action understanding, our attempt to operationalize this more precise distinction may be a useful starting point for further research.

## Conflict of interest statement

The authors declare that the research was conducted in the absence of any commercial or financial relationships that could be construed as a potential conflict of interest.

## References

[B1] Catmur C.. (2014). Unconvincing support for role of mirror neurons in “action understanding”: commentary on Michael Front. Hum. Neurosci. 8:553. 10.3389/fnhum.2014.00553 25120455 PMC4110945

[B3] Hamilton A. Grafton S. (2007). The motor hierarchy: from kinematics to goals and intentions, in Sensorimotor Foundations of Higher Cognition: Attention and Performance eds Haggard P. Rossetti Y. Kawato M. (Oxford, England: Oxford University Press), 381–408

[B4] Michael J. Sandberg K. Skewes J. Wolf T. Blicher J. Overgaard M.. (2014). TMS (cTBS) demonstrates a causal role of premotor homunculus in action understanding. Psychol. Sci. 25, 963–972. 10.1177/0956797613520608 24549297 PMC4232279

